# Effect of Continuous Intraoperative Dexmedetomidine on Interleukin-6 and Other Inflammatory Markers After Coronary Artery Bypass Graft Surgery: A Randomized Controlled Trial

**DOI:** 10.3390/medicina61050787

**Published:** 2025-04-24

**Authors:** Ranko Zdravković, Sanja Vicković, Mihaela Preveden, Vanja Drobnjak, Mirka Lukić-Šarkanović, Iva Bosić Miljević, Milanka Tatić, Teodora Tubić, Nebojša Videnović, Nikola Mladenović, Nikola Komazec, Nina Dračina, Milica Jerković, Aleksandra Djoković, Aleksandar Jakovljević, Arijeta Kostić, Erdin Mehmedi, Aleksandar Redžek

**Affiliations:** 1 Faculty of Medicine, University of Novi Sad, 21000 Novi Sad, Serbia; 2 Institute of Cardiovascular Diseases of Vojvodina, 21208 Sremska Kamenica, Serbia; 3 Clinical Center of Vojvodina, 21000 Novi Sad, Serbia; 4 Institute of Oncology of Vojvodina, 21204 Sremska Kamenica, Serbia; 5 Faculty of Medicine, University of Pristina, 38220 Kosovska Mitrovica, Serbia; 6 Clinic of Internal Medicine, Clinical Hospital Center Kosovska Mitrovica, 38220 Kosovska Mitrovica, Serbia

**Keywords:** dexmedetomidine, cardiopulmonary bypass, inflammatory response

## Abstract

*Background and Objectives:* Coronary artery bypass graft (CABG) surgery is the most common cardiac surgery. One of the main causes of postoperative complications and increased mortality after CABG is the inflammatory response. The aim of this study was to investigate whether continuous intraoperative dexmedetomidine can reduce the increase of IL-6 and other inflammatory markers after CABG surgery. *Materials and Methods:* The study is registered with ClinicalTrials.gov, NCT06378827, accessed on 23 April 2024. This prospective experimental study was conducted from April to December 2024 and included 100 patients undergoing CABG surgery. Patients in the experimental group (50 patients) received a continuous infusion of dexmedetomidine (0.5 μg/kg/h) from anesthesia induction until the end of surgery, while the patients in the control group (50 patients) received the same volume of saline. The primary outcomes were the changes in the values of interleukin-6 (IL-6), C-reactive protein (CRP), white blood cells (WBC), and fibrinogen on the first postoperative day (POD1) compared to the basal, preoperative values. *Results:* The patients in the control group were on average 65.26 years old, and the patients in the experimental group were 66.28 years old (*p* = 0.555). From the control group, 40 (80%) patients were male compared to 37 (74%) patients from the experimental group (*p* = 0.635). Median IL-6 before surgery was 2.0 pg/mL, while on POD 1 it was 76.2 pg/mL (*p* < 0.001). Median CRP before surgery was 2.5 mg/dL, while the POD1 value was 45.5 mg/dL (*p* < 0.001). Median WBC values were 6.7 × 10^9^/L before surgery and 13.6 × 10^9^/L on POD1 (*p* < 0.001). The average value of fibrinogen was 3.19 g/L before surgery, while on POD1 it was 3.37 g/L (*p* = 0.024). The increase in IL-6 on POD1 (ΔIL-6) was 72.4 pg/mL in the control group and 73.0 pg/mL in the experimental group (*p* = 0.427). ΔCRP was 41.2 mg/mL (control group) and 38.0 mg/mL (experimental group) (*p* = 0.725). ΔWBC was 7.45 × 10^9^/L (control group) and 6.81 × 10^9^/L (experimental group) (*p* = 0.407). Δfibrinogen was 0.16 g/L (control group) and 0.2 g/L (experimental group) (*p* = 0.771). *Conclusions:* Intraoperative administration of dexmedetodine during CABG surgery at a dose of 0.5 µg/kg/h without a loading dose does not lead to a decrease in the intensity of the inflammatory response after surgery.

## 1. Introduction

Coronary artery disease (CAD) is a cardiovascular disease that has been found to be the leading cause of death in both developed and developing countries. About 30–40% of annual deaths have been estimated to be related to CAD [1]. CAD is a cardiovascular disorder that occurs due to atherosclerosis or atherosclerotic occlusions of the coronary arteries. The risk factors associated with the development of CAD have been established by extensive epidemiological research, and these are smoking, diabetes, hyperlipidemia, and hypertension [1]. Globally, the prevalence of CAD increases significantly with age. The main indications for coronary artery bypass graft (CABG) surgery are three-vessel coronary artery disease, 50% stenosis of the left main coronary artery, two-vessel coronary artery disease if the anterior descending artery is significantly narrowed, and single-vessel coronary artery disease if the patient has pronounced anginal symptoms despite adequate medical therapy or impossible percutaneous coronary intervention [2]. CABG is still the most commonly performed cardiac surgery procedure worldwide, representing annual volumes of approximately 200,000 isolated cases in the US and an average incidence rate of 62 per 100,000 inhabitants in Western European countries [3]. Over the last few decades, mortality after this major surgery has decreased, and now the 30-day mortality is 2% [4,5]. However, postoperative complications are still extremely common. Complications following CABG can result in adverse outcomes and death and increase intensive care unit (ICU) admissions and the length of stay [6,7,8].

One of the main causes of postoperative complications and increased mortality after CABG, especially in surgery performed with cardiopulmonary bypass (CPB), is the inflammatory response associated with major surgery and blood contact with the artificial CPB system [9,10]. While localized inflammation is important for tissue repair and wound healing, the systemic inflammatory response, if dysregulated, can lead to organ damage and, in the most severe cases, to death [11,12]. Interleukin-6 (IL-6) is a central pro-inflammatory cytokine and a key marker of the inflammatory response during CPB. Elevated IL-6 levels correlate with postoperative complications, including impaired lung function, circulatory instability, and organ dysfunction [13]. Unfortunately, IL-6 analysis is not a routine laboratory procedure in many centers. It is necessary to analyze correlations between IL-6 and inflammatory markers in routine clinical use to find out which inflammatory marker correlates best with IL-6. C-reactive protein (CRP) is an acute phase protein and is used as a marker for tissue damage and inflammation [14]. White blood cells (WBCs) are the primary effectors of the immune system protecting the body against infection and resolving tissue injury [15]. Elevated WBC count is a cardinal sign of acute inflammation, but simple elevation in WBC count is highly non-specific, and the rates of change and resolution in WBCs associated with favorable acute inflammatory responses are not well-defined [16]. Fibrinogen, a clotting factor, acts as a positive acute phase reactant, which is increased during an inflammatory response [17].

Dexmedetomidine, a highly selective α2 adrenergic receptor agonist, is increasingly used perioperatively for its analgesic, sedative, anxiolytic, and sympatholytic properties [18,19,20,21]. The advantages of using dexmedetomidine in perioperative cardiac surgical care are well established, but almost all the studies concentrate on its use as a sedative in the postoperative period. On the other hand, there are not many studies that have examined the intraoperative use of dexmedetomidine as an adjuvant to general anesthesia. The aim of this study was to examine whether intraoperative continuous administration of dexmedetomidine during CABG leads to a reduced increase in IL-6 and other inflammatory markers and the correlations between IL-6 and inflammatory markers most commonly used in clinical practice.

## 2. Materials and Methods

### 2.1. Study Population

The study is registered with ClinicalTrials.gov, NCT06378827, accessed on 23 April 2024. The study was conducted at the Clinic for Cardiovascular Surgery of the Institute of Cardiovascular Diseases of Vojvodina. The study protocol is in accordance with the Helsinki Declaration, and the study was started after obtaining the approval of the Ethics Committee of the Institute of Cardiovascular Diseases of Vojvodina (protocol code 418-1/2, date of approval 11 March 2024). All included patients signed an informed consent prior to surgery. The inclusion criteria were as follows: patients older than 18 years scheduled for CABG under CPB, American Society of Anesthesiologists (ASA) physical classification grade III-IV, and New York Heart Association functional class grade ≤ III. The exclusion criteria were as follows: emergency CABG, off-pump CABG, left ventricular ejection fraction ≤ 30%, concomitant valve surgery with CABG, severe kidney or liver dysfunction, second- and third-degree atrioventricular block, allergy to dexmedetomidine, and patients who received anti-inflammatory drugs.

### 2.2. Randomisation

This was a prospective, randomized, controlled trial. All patients included in the study were randomized in a 1:1 ratio, using computer-generated numbers, into two groups. The participants, the doctors who participated in the preoperative preparation of the patients, surgeons, doctors in the ICU, laboratory technicians, and others who participated in postoperative treatment did not know into which group the patients were randomized.

### 2.3. Anesthesia Management

Patients underwent routine preoperative fasting. Anesthesia was conducted according to the same protocol in both groups. After adequate preoxygenation, intravenous induction of anesthesia was performed using midazolam (0.04–0.05 mg/kg), sufentanil (0.1–0.15 μg/kg), propofol (1 mg/kg), and rocuronium bromide (0.8 mg/kg). Anesthesia was maintained with sevoflurane (with a minimum alveolar concentration of 0.5–1). A continuous infusion of sufentanil (0.4–1 μg/kg/h) was used for intraoperative analgesia. Neuromuscular relaxation was maintained by intermittent administration of bolus doses of rocuronium bromide. All patients received 2 g of tranexamic acid. For antibiotic prophylaxis, cefazolin (1 g) was used half an hour before surgery. The heart rate and blood pressure were maintained within 20% of the baseline values. If there was a need, we included vasopressors or inotropic support. After surgery, the patients were transferred to the cardiac surgery ICU. Sedation was discontinued when hemodynamic and rhythmic stability, adequate metabolic status, adequate gas exchange, normothermia, and the absence of significant thoracic drainage were achieved. The patient’s readiness to be weaned from mechanical lung ventilation (MLV) was assessed based on the following parameters: respiratory rate of ≤30 breaths/min, maximum inspiratory pressure of ≤−25 cm H_2_O, tidal volume of >5 mL/kg, rapid shallow breathing index of 105 breaths/min/L, and adequate oxygenation (SpO_2_ > 90%) at inspiratory fraction of oxygen (FiO_2_) ≤ 0.4 or PaO_2_/FiO_2_ ≥ 150 mmHg. After weaning, a spontaneous breathing test (SBT) was performed, usually using an automatic tube compensation that was adjusted for the assumed tube resistance for about 30 min. After a successful SBT, the patient was extubated.

### 2.4. Surgery and CPB Management

All operations were performed through a medial sternotomy. To achieve total heparinization (activated clotting time > 480 s), heparin was given in a dose of 300–400 IU/kg. A membrane oxygenator was used for CPB, which was previously primed with 1500 mL of Ringer’s lactate solution and 2500 IU of heparin. The flow rate of the non-pulsatile pump was 2–2.4 L/min/m^2^. All operations were performed under conditions of mild hypothermia (33–34 °C). Mean arterial pressure during CPB was maintained between 50 and 80 mmHg.

### 2.5. Study Interventions

Patients in the experimental group received a continuous infusion of dexmedetomidine (0.5 μg/kg/h) from anesthesia induction until the end of surgery. The dexmedetomidine solution was prepared by diluting one ampoule (200 μg/2 mL) with 48 mL of saline to obtain a 4 µg/mL solution. Patients in the control group were infused with an equal volume of saline. A study examining the effect of different doses of continuous infusion of dexmedetomidine showed that a dose of 0.5 µg/kg/h can maintain the stability of cardiac electro-physiology during the perioperative period and has no significant adverse effects on cardiac circulation efficiency [22]. This is the reason why we decided on the given dose of dexmedetomidine.

### 2.6. Outcomes

The primary outcomes were the changes in the values of IL-6, CRP, WBC count, and fibrinogen on the first postoperative day (POD1) compared to the basal, preoperative values. IL-6 analyses were performed on the AFIAS-1 apparatus (Chuncheon-Si, Republic of Korea). Here, we must mention that the device did not calculate values below 2 pg/mL, so we calculated the values in those patients as 2 pg/mL. Other analyses were performed on a Beckman Coulter AU 480 (Brea, CA, USA).

The secondary outcomes were the correlations between IL-6 and other inflammatory markers used in clinical practice.

### 2.7. Statistical Analysis

Using G*power software (v. 3.1.9.6, Franz Faul, University of Kiel, Germany) and data from the literature [23], we calculated the needed sample size with an I type error probability of α = 0.05 and a study power of 0.8. The minimal number of patients to be included, with the 1:1 ratio between the experimental and control groups, was 40 (20 per group). In order to increase the power of the study, we decided to include 100 patients (50 per group). The obtained data were analyzed using the statistical package Statistical Package for Social Sciences (SPSS v.25, Chicago, IL, USA). Continuous variables are presented as a mean value with a standard deviation or median with interquartile range (depending on the normality of the distribution), while categorical variables are presented as absolute and relative values with frequency. The normality of the distribution was determined based on a set of properties of the distribution (symmetry, flatness, presence of extreme values, and a Shapiro–Wilk test). Experimental and control groups were compared using parametric (Student’s *t*-test) and non-parametric tests (Mann–Whitney U-test and χ^2^-test). Correlations between inflammatory markers were analyzed based on Spearman’s correlation coefficient. A *p* value < 0.05 was considered statistically significant.

## 3. Results

**Figure 1:**
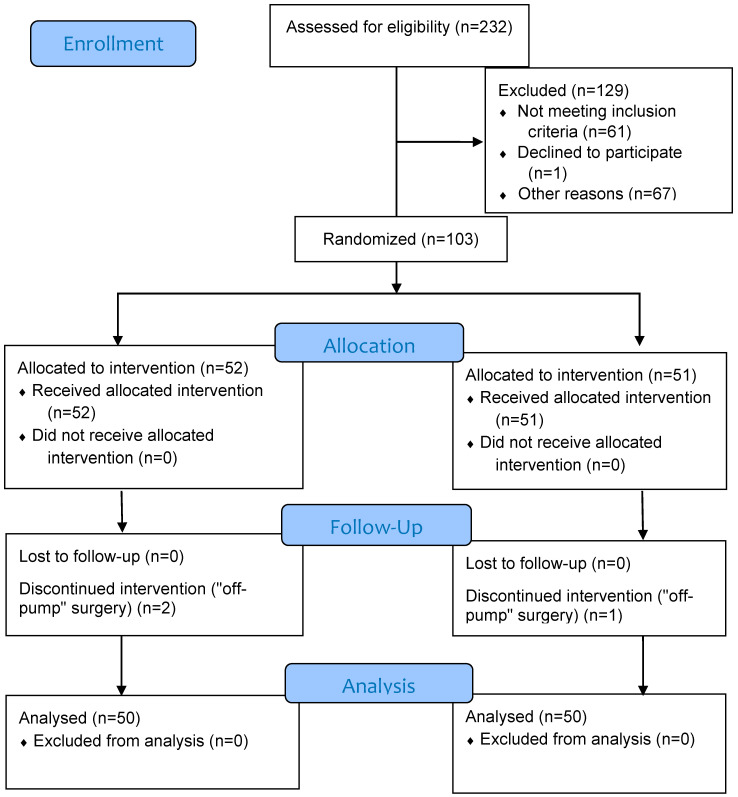
CONSORT flow diagram.

After excluding patients who did not meet the criteria for the study, 103 patients were included in the study and divided into two groups. Of these patients, three patients were excluded from the study because “off-pump” surgery was performed. Finally, 100 patients were analyzed, 50 in each group (Figure 1).

The general characteristics of the patients did not differ between the groups, thus excluding the influence of these characteristics on the primary outcome (Table 1).

Table 2 shows the comorbidities of the patients. There were no statistically significant differences in comorbidities between groups.

Chronic cardiology therapy was equally represented in both groups (Table 3).

The duration of surgery, cross-clamp time, and CPB time were almost equal between the groups (Table 4). The median of bypass grafts in both groups was 2.0 (2.0–3.0).

The dose of sufentanil administered to patients in the control group was, on average, 185 µg, while in the experimental group, it was significantly lower and amounted to 107.5 µg (*p* < 0.001) (Table 4). Patients in the experimental group also received significantly less sevoflurane (15.13 ± 6.79 mL vs. 20.81 ± 4.40 mL; *p* < 0.001). The use of vasopressors during surgery was more common in patients in the experimental group, but the difference was not statistically significant. On the other hand, the use of inotropes was more frequent in patients in the control group (30 vs. 20, *p* = 0.029). The use of crystalloid solutions and red blood cells was equal in both groups.

The groups did not differ in duration of MLV, ICU stay, hospital length of stay, and in-hospital mortality (Table 4).

Table 5 shows inflammatory markers the morning before surgery and on POD1, as well as the change (∆) during that period. Median IL-6 before surgery was 2.0 pg/mL, while on POD 1, it was 76.2 pg/mL (*p* < 0.001). The median CRP before surgery was 2.5 mg/dL, while the POD1 value was 45.5 mg/dL (*p* < 0.001). Median WBC values were 6.7 × 10^9^/L before surgery and 13.6 × 10^9^/L on POD1 (*p* < 0.001). The average value of fibrinogen was 3.19 g/L before surgery, while on POD1, it was 3.37 g/L (*p* = 0.024).

However, the difference in the increase of inflammatory markers did not differ between groups, as shown in Table 6. The value of IL-6 on POD1 was 72.4 pg/mL higher than the pre-operative value in the control group, while in the experimental group, the difference was 73.0 pg/mL (*p* = 0.427). The increase in CRP was 41.2 mg/dl in the control group and 38.0 mg/dL in the experimental group (*p* = 0.725). Δ WBC was 7.45 × 10^9^/L in the control group versus 6.81 × 10^9^/L in the experimental group (*p* = 407). The increase in fibrinogen was 0.16 g/L in the control group and 0.20 in the experimental group (*p* = 0.771).

**Figure 2:**
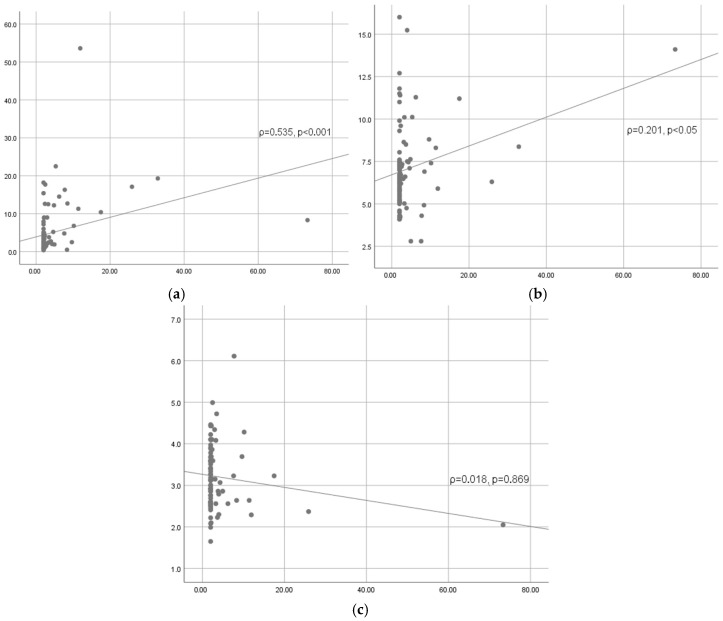
Correlations between IL-6 and other inflammatory markers before surgery: (**a**) correlation between IL-6 and CRP, (**b**) correlation between IL-6 and WBC, and (**c**) correlation between IL-6 and fibrinogen.

The correlations between IL-6 and other inflammatory markers before surgery are shown in Table 7 and Figure 2. Spearman’s coefficient ρ = 0.535 indicates a moderate positive correlation between IL-6 and CRP (*p* < 0.001). There is a weak positive correlation between the preoperative values of IL-6 and WBC (ρ = 0.201, *p* < 0.05), while there is no significant correlation between IL-6 and fibrinogen (ρ = 0.018, *p* = 0.869).

**Figure 3:**
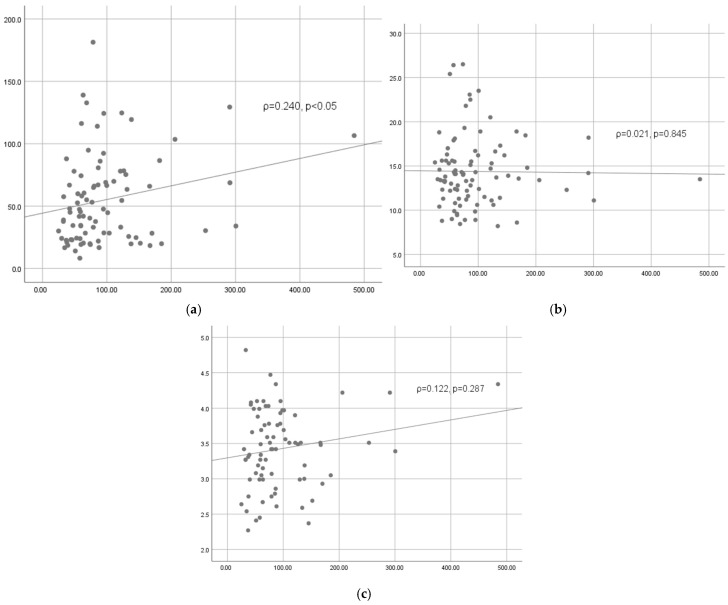
Correlations between IL-6 and other inflammatory markers on the first postoperative day: (**a**) correlation between IL-6 and CRP, (**b**) correlation between IL-6 and WBC, and (**c**) correlation between IL-6 and fibrinogen.

In general, the correlations between IL-6 and other inflammatory markers in POD1 are weaker (Figure 3). There is a weak positive correlation between IL-6 and CRP (ρ = 0.240, *p* < 0.05). The correlations between IL-6 and WBC (ρ = 0.021, *p* = 0.845) and between IL-6 and fibrinogen (ρ = 0.122, *p* = 0.287) are not significant.

## 4. Discussion

Given that dexmedetomidine has anti-inflammatory properties, our idea was to examine whether its continuous intraoperative administration during CABG, at a dose of 0.5 µg/kg/h without a loading dose, could lead to a lower increase in IL-6 and other inflammatory markers. Our study showed that the values of IL-6 and other inflammatory markers increase significantly after CABG, which indicates a very pronounced inflammatory response to surgical trauma and CPB. However, there was no difference in the increase when comparing the dexmedetomidine-administered group and the control group.

For this study, we did not apply a loading dose (0.5–1 µg/kg for 10 min) for two reasons. The first is that we included dexmedetomidine after the induction of anesthesia. Another reason is the hemodynamic and rhythmic instability that a loading dose can lead to. Given that dexmedetomidine has analgesic and sedative effects, it was expected that patients receiving it during surgery would have less need for opioid analgesics and anesthetics. This was proven in our study. Patients from the experimental group received significantly lower doses of sufentanil and sevoflurane. However, despite a significantly lower dose of opioid analgesic during surgery, this did not affect the shortening of postoperative MLV time compared to the control group. Dexmedetomidine may cause bradycardia and hypotension. Our study showed that it is safe at a dose of 0.5 µg/kg/h. There were no adverse events. Moreover, the use of inotropes was significantly less frequent in patients of the experimental group. On the other hand, there was no significant difference in the need for a vasopressor.

CABG is the standard of care for the treatment of advanced coronary artery disease [24]. Despite the improvement of surgical and anesthesiology techniques, as well as CPB devices, the inflammatory response after cardiac surgery is still very pronounced [10,25]. Inflammatory response, initiated by monocytes and macrophages at the site of the injury, involves a complex biochemical cascade that includes the release of cytokines such as IL-6, tumor necrosis factor-α (TNF-α), and IL-1β [26]. Among these inflammatory markers, IL-6 serves as a primary proinflammatory cytokine that is produced within 2–4 h after a tissue injury, making it an early and valuable independent marker [25,27]. A recent study found IL-6 to be a good predictor of postoperative complications and outcomes in cardiac surgery patients, where the majority were patients undergoing CABG surgery [28]. A large meta-analysis also found an association between IL-6 and CRP and postoperative complications after cardiac surgery [29]. It is known that increased IL-6 levels in patients monitored in the ICU are associated with a poor prognosis [25,30,31].

A meta-analysis by Wang et al., which included 37 studies examining the association between dexmedetomidine and IL-6, showed that continuous administration of dexmedetomidine was associated with lower IL-6 levels [18]. A study investigating the effect of perioperative administration of dexmedetomidine (0.6 μg/kg/h of dexmedetomidine intravenously from 10 min before anesthesia induction to 6 h after surgery) on renal function after valvular cardiac surgery found a lower incidence of acute kidney injury in the dexmedetomidine group [32]. Those patients had lower values of CRP, IL-6, and TNF-α immediately after the operation as well as 24 h later. A study examining the impact of intraoperative administration of dexmedetomidine in patients undergoing surgery for infective endocarditis did not show positive results regarding the reduction of inflammatory markers, including CRP, WBC, neutrophils, and IL-6, on POD1 [33]. One of the explanations for such results was that the inflammatory process was already in serious progress due to the pathology of the disease itself and that the potential anti-inflammatory effect can be expressed if it is activated before the inflammatory response occurs. A study examining the effect of continuous infusion of dexmedetomidine during laparoscopic hysterectomy at a dose of 0.4 µg/kg/h did not indicate a decrease in IL-6 values compared to the control group [34]. On the other hand, a randomized study in which a dose of 0.5 µg/kg/h was used in elderly patients undergoing thoracolumbar compression fracture surgery showed lower concentrations of IL-6 after surgery [35]. It is concluded that dexmedetomidine can have an anti-inflammatory effect in higher doses and when it is administered before the onset of the inflammatory response, as well as when its administration is extended later in the postoperative period. However, with an increase in the dose and prolongation of its administration, its most common side effects, bradycardia and hypotension, can be expected. Interestingly, a study that investigated the effect of a lower dose of dexmedetomidine (0.3 µg/kg/h) during cardiac surgery with the use of mini-CPB found a lower increase in the inflammatory markers IL-1, IL-6, and TNF-α compared to the group of patients who did not receive dexmedetomidine [36]. However, this was a small study with only 23 patients.

CRP is primarily produced by the liver in response to IL-6 and shows a good correlation with IL-6 levels [27]. Our study showed that CRP values also increase several tens of times compared to preoperative values. The WBC values are twice as high compared to the basal preoperative values, while there is also a noticeable increase in fibrinogen. However, as with IL-6, there was no difference in the increase between groups for these inflammatory markers. A double-blind randomized controlled trial that investigated the effect of administering dexmedetomidine during laparoscopy-assisted gastrectomy at a dose of 0.5 µg/kg/h did not determine a difference in the increase of CRP and WBC, but it did in the values of IL-6, which were lower in the group with dexmedetomidine. A study of septic patients requiring MLV found that patients sedated with dexmedetomidine showed lower CRP values than patients sedated with other drugs [37].

Given that the analysis of IL-6 is not a routine procedure at our clinic nor in most centers in our country, our idea was to determine the correlation between IL-6 and the inflammatory markers that we most often use in clinical practice, which would help us in our further work and the assessment of the intensity of the inflammatory response. The strongest correlation was seen in comparison with CRP. A cross-sectional study examining the correlation between IL-6 and CRP showed a similar result to our study; the Pearson correlation coefficient (r) was 0.61, which indicates a strong positive correlation [38]. A study examining the correlation between IL-6 and WBC counts showed a moderate positive correlation between IL-6 and WBC [39]. This correlation, however, in our study is weaker but still positive. Although Cronjé et al. [40] showed a strong correlation between IL-6 and fibrinogen in their study, our study showed that there is no significant correlation between these two inflammatory markers.

### Limitations of the Study

There were several limitations to this study. First, the apparatus on which we analyzed IL-6 values did not measure values less than 2 pg/mL, so we calculated the values in those patients as 2 pg/mL. However, considering that the postoperative values are several tens of times higher than the preoperative ones, and on average, they are over 70 pg/mL, we believe that this small limitation does not affect the final results. Second, four inflammatory markers were examined. Some future studies could include a larger number of inflammatory markers, including markers such as TNF-α or IL-10. Third, this is a single-center study. For better validation of the results, it is necessary to conduct multicenter studies with a larger number of patients.

## 5. Conclusions

Continuous intraoperative administration of dexmedetomidine during CABG surgery, at a dose of 0.5 µg/kg/h without a loading dose, does not lead to a lessened increase in IL-6 or other inflammatory markers on the first postoperative day. Preoperatively, there is a moderate positive correlation between IL-6 and CRP and a weak positive correlation between IL-6 and WBC counts, while postoperatively, only the correlation between IL-6 and CRP is significant.

At a dose of 0.5 mg, dexmedetomidine is safe and has not led to adverse events. For a better effect of dexmedetomidine on the inflammatory response after CABG, it would be desirable to consider administering a loading dose, as well as longer-term administration after surgery. Also, the use of a higher dose than 0.5 µg/kg/h should be considered.

## Figures and Tables

**Table 1:** Patient characteristics.

Variables	Control Group (*n* = 50)	Experimental Group (*n* = 50)	*p*
Age, years	65.26 ± 9.04	66.28 ± 8.18	0.555
Gender			
Male, *n* (%)	40 (80)	37 (74)	0.635
Female, *n* (%)	10 (20)	13 (26)	
Height, cm	171.73 ± 9.00	169.41 ± 9.47	0.214
Weight, kg	80.0 (75.0–90.5)	76.8 (66.6–91.2)	0.090
BMI, kg/m^2^	28.24 ± 4.38	27.22 ± 5.13	0.294
LVEF, %	55.06 ± 7.06	54.18 ± 7.14	0.537
ASA classification			
III, *n* (%)	36 (72)	32 (64)	0.521
IV, *n* (%)	14 (28)	18 (36)	
EuroScore II	0.9 (0.8–1.4)	1.0 (0.7–1.5)	0.903

Values are presented as mean ± SD, median (Q1, Q3), or number (%). BMI—body mass index; LVEF—left ventricular ejection fraction; ASA—American Society of Anesthesiologists.

**Table 2:** Comorbidities.

Variables	Control Group (*n* = 50)	Experimental Group (*n* = 50)	*p*
Hypertension, *n* (%)	49 (98)	49 (98)	1.000
History of MI, *n* (%)	21 (42)	22 (44)	1.000
Atrial fibrillation, *n* (%)	3 (6)	5 (10)	0.715
Diabetes mellitus, *n* (%)	17 (34)	18 (36)	1.000
History of stroke, *n* (%)	3 (6)	1 (2)	0.617
COPD, *n* (%)	7 (14)	6 (12)	1.000
Anemia, *n* (%)	1 (2)	3 (6)	0.617
Dyslipidemia, *n* (%)	43 (86)	45 (90)	0.760
Smoking, *n* (%)	21 (42)	19 (38)	0.838

MI—myocardial infarction; COPD—chronic obstructive pulmonary disease.

**Table 3:** Chronic therapy.

Variables	Control Group (*n* = 50)	Experimental Group (*n* = 50)	*p*
Beta blockers, *n* (%)	42 (84)	43 (86)	1.000
ACEi/ARBs, *n* (%)	35 (70)	34 (68)	1.000
CCBs, *n* (%)	15 (30)	22 (44)	0.214
Diuretics, *n* (%)	23 (46)	25 (50)	0.841
Antiplatelets, *n* (%)	38 (76)	33 (66)	0.378
Anticoagulants, *n* (%)	1 (2)	2 (4)	1.000

ACEi/ARBs—Angiotensin-converting enzyme (ACE) inhibitors/Angiotensin receptor blockers; CCBs—calcium channel blockers.

**Table 4:** Intraoperative and postoperative data.

Variables	Control Group (*n* = 50)	Experimental Group (*n* = 50)	*p*
Duration of surgery, min	167.24 ± 31.71	169.80 ± 30.70	0.685
Cross-clamp time, min	51.54 ± 21.35	48.90 ± 17.72	0.503
CPB time, min	58.08 ± 21.66	56.58 ± 18.69	0.712
Number of bypass grafts, *n*	2.0 (2.0–3.0)	2.0 (2.0–3.0)	0.898
Sufentanil, µg	185.0 (152.5–232.5)	107.5 (90.8–132.5)	<0.001
Sevoflurane, mL	20.81 ± 4.40	15.13 ± 6.79	<0.001
Vasopressors, *n* (%)	16 (32)	22 (44)	0.303
Inotropes, *n* (%)	30 (60)	20 (40)	0.029
Crystalloids, mL	1454.08 ± 233.59	1408.0 ± 333.71	0.427
Red blood cells, *n* (%)	4 (8)	5 (10)	1.000
Duration of MLV, hours	7.0 (5.0–8.2)	7.0 (5.0–8.2)	0.702
ICU stay, days	1.0 (1.0–1.0)	1.0 (1.0–1.0)	0.651
Hospital LOS, days	6.0 (5.0–7.0)	6.0 (5.0–7.2)	0.761
In-hospital mortality, *n* (%)	0 (0)	1 (2)	1.000

Values are presented as mean ± SD, median (Q1, Q3), or number (%). CPB—cardiopulmonary bypass; MLV—mechanical lung ventilation; ICU—intensive care unit; LOS—length of stay.

**Table 5:** Inflammatory markers.

Variables	Before Surgery	POD1	∆	*p*
IL-6, pg/mL	2.0 (2.0–3.3)	76.2 (54.9–121.1)	73.7 (50.8–113.2)	<0.001
CRP, mg/dL	2.5 (1.2–5.2)	45.5 (24.5–72.0)	38.8 (24.0–66.7)	<0.001
WBC, (×10^9^/L)	6.7 (5.6–7.5)	13.6 (11.3–15.6)	7.0 (4.5–9.6)	<0.001
Fibrinogen, g/L	3.19 ± 0.78	3.37 ± 0.52	0.18 ± 0.69	0.024

Values are presented as mean ± SD or median (Q1, Q3). POD1—the first postoperative day; IL-6—interleukin-6; CRP—C-reactive protein; WBC—white blood cells.

**Table 6:** Difference in inflammatory markers between groups.

Variables	Control Group (*n* = 50)	Experimental Group (*n* = 50)	*p*
IL-6, pg/mL			
Before surgery	2.0 (2.0–2.5)	2.0 (2.0–4.2)	0.446
POD1	77.0 (47.3–103.8)	75.0 (55.8–125.1)	0.408
Δ IL-6	72.4 (41.9–101.8)	73.0 (51.0–116.9)	0.427
CRP, mg/dL			
Before surgery	2.5 (1.2–4.1)	2.4 (1.1–8.9)	0.690
POD1	45.5 (24.1–68.7)	46.3 (24.6–75.6)	0.979
Δ CRP	41.2 (24.8–67.5)	38.0 (23.2–69.6)	0.725
WBC, ×10^9^/L			
Before surgery	6.8 (5.6–7.4)	6.5 (5.4–7.8)	0.772
POD1	14.1 (12.2–16.3)	13.3 (11.0–15.38)	0.198
Δ WBC	7.45 ± 3.55	6.81 ± 4.05	0.407
Fibrinogen, g/L			
Before surgery	3.26 ± 0.74	3.20 ± 0.83	0.714
POD1	3.53 ± 0.58	3.38 ± 0.57	0.230
Δ Fibrinogen	0.16 ± 0.53	0.20 ± 0.80	0.771

Values are presented as mean ± SD or median (Q1, Q3). IL-6—interleukin-6; POD1—the first postoperative day; CRP—C-reactive protein; WBC—white blood cells.

**Table 7:** Correlations between IL-6 and other inflammatory markers.

	IL-6 Before Surgery [ρ (*p*)]	IL-6 POD1 [ρ (*p*)]
CRP before surgery	0.535 (<0.001)	
CRP POD1		0.240 (<0.05)
WBC before surgery	0.201 (<0.05)	
WBC POD1		0.021 (0.845)
Fibrinogen before surgery	0.018 (0.869)	
Fibrinogen POD1		0.122 (0.287)

WBC—white blood cells; POD1—the first postoperative day.

## Data Availability

All data are available from the corresponding author upon reasonable request.
